# The Relationship of Duffy Gene Polymorphism with High-Sensitivity C-Reactive Protein, Mortality, and Cardiovascular Outcomes in Black Individuals

**DOI:** 10.3390/genes15111382

**Published:** 2024-10-27

**Authors:** Edward T. Ha, Jeffery Haessler, Kent D. Taylor, Bjoernar Tuftin, Matt Briggs, Manish A. Parikh, Stephen J. Peterson, Robert E. Gerszten, James G. Wilson, Karl Kelsey, Usman A. Tahir, Teresa Seeman, Stephen S. Rich, April P. Carson, Wendy S. Post, Charles Kooperberg, Jerome I. Rotter, Laura M. Raffield, Paul Auer, Alex P. Reiner

**Affiliations:** 1 Division of Cardiology, Department of Internal Medicine, NewYork-Presbyterian Brooklyn Methodist Hospital, Brooklyn, NY 11215, USA; mattstat@gmail.com (M.B.); map2007@nyp.org (M.A.P.); stp9039@nyp.org (S.J.P.); 2 Division of Public Health Sciences, Fred Hutchinson Cancer Center, Seattle, WA 98109, USA; jhaessle@whi.org (J.H.); clk@fredhutch.org (C.K.); 3 The Institute for Translational Genomics and Population Sciences, The Lundquist Institute of Biomedical Innovation at Harbor-UCLA Medical Center, Torrance, CA 90502, USA; ktaylor@lundquist.org (K.D.T.); jrotter@lundquist.org (J.I.R.); 4 Department of Genetics, University of North Carolina at Chapel Hill, Chapel Hill, NC 27599, USA; btuftin@email.unc.edu (B.T.); laura_raffield@unc.edu (L.M.R.); 5 Department of Medicine, Weill Cornell Medical College, New York, NY 10065, USA; 6 Division of Cardiovascular Medicine, Beth Israel Deaconess Medical Center, Harvard Medical School, Boston, MA 02215, USA; rgerszte@bidmc.harvard.edu (R.E.G.); jwilson8@bidmc.harvard.edu (J.G.W.); utahir@bidmc.harvard.edu (U.A.T.); 7 Broad Institute of Harvard and MIT, Cambridge, MA 02142, USA; 8 Department of Epidemiology and Pathology and Laboratory Medicine, Brown University, Providence, RI 02903, USA; karl_kelsey@brown.edu; 9 Department of Medicine, David Geffen School of Medicine at UCLA, Los Angeles, CA 90095, USA; tseeman@mednet.ucla.edu; 10 Center for Public Health Genomics, Department of Public Health Sciences, University of Virginia School of Medicine, Charlottesville, VA 22908, USA; ssr4n@virginia.edu; 11 Department of Medicine, University of Mississippi Medical Center, Jackson, MS 39216, USA; apcarson@umc.edu; 12 Division of Cardiology, Department of Medicine, Johns Hopkins University School of Medicine, Baltimore, MD 21287, USA; wpost@jhmi.edu; 13 Division of Biostatistics and Institute for Health and Equity, Medical College of Wisconsin, Milwaukee, WI 53226, USA; pauer@mcw.edu; 14 Department of Epidemiology, University of Washington, Seattle, WA 98195, USA; apreiner@uw.edu

**Keywords:** Duffy receptor for chemokines, atypical chemokine receptor 1, healthcare disparity, hs-CRP, Duffy gene polymorphism

## Abstract

**Background:** Black adults have higher incidence of all-cause mortality and worse cardiovascular disease (CVD) outcomes when compared to other U.S. populations. The Duffy chemokine receptor is not expressed on erythrocytes in a large majority of Black adults, but the clinical implications of this are unclear. **Methods:** Here, we investigated the relationship of Duffy receptor status, high-sensitivity C-reactive protein (hs-CRP), and mortality and incident CVD events (coronary heart disease, stroke, and heart failure) in self-identified Black members of three contemporary, longitudinal cohort studies (the Women’s Health Initiative, Jackson Heart Study, and Multi-Ethnic Study of Atherosclerosis). Data on 14,358 Black participants (9023 Duffy-null and 5335 Duffy-receptor-positive, as defined using single-nucleotide polymorphism (SNP) rs2814778) were included in this analysis. **Results:** Duffy null was strongly associated with higher hs-CRP (meta-analysis *p* = 2.62 × 10^−9^), but the association was largely attenuated, though still marginally significant (*p* = 0.005), after conditioning on known *CRP* locus alleles in linkage disequilibrium with the Duffy gene. In our discovery cohorts, Duffy-null status appeared to be associated with a higher risk of all-cause mortality and incident stroke, though these associations were attenuated and non-significant following adjustment for traditional risk factors including hs-CRP. Moreover, the association of Duffy-null status with mortality could not be replicated in an independent sample of Black adults from the UK Biobank. **Conclusions:** These findings suggest that the higher levels of hs-CRP found in Duffy-null individuals may be in part independent of CRP alleles known to influence circulating levels of hs-CRP. During the follow-up of this community-based sample of Black participants, Duffy-null status was not associated with mortality or incident CVD events independently of traditional risk factors including hs-CRP.

## 1. Introduction

In the United States, Black individuals have a particularly high burden of mortality and adverse cardiovascular disease outcomes, which the current literature suggests is largely due to adverse social determinants of health-related exposures [1]. Black populations traditionally have been understudied in cardiovascular genetics and epidemiologic analyses, and further assessment of putative health risk factors is needed in this population [2]. Most individuals of West African descent are homozygous for a single-nucleotide polymorphism (SNP) (T > C mutation at nucleotide −33) in the *DARC* or *ACKR1* gene promoter region that disrupts a GATA-1 binding site, leading to erythroid-specific loss of expression of the Duffy antigen for chemokines (Duffy null) and protection from malaria [3]. The Duffy-null mutation is also prevalent in the Middle East and in other populations with significant African ancestry such as Hispanics/Latinos but extremely rare in other populations with ancestry from regions in which malaria is non-endemic [4,5]. Initially identified as a contributor to the benign phenotype of “ethnic” neutropenia, through its functioning as an atypical chemokine receptor, the loss of the Duffy receptor on erythrocytes may modulate circulating chemokine/cytokine levels and host response to inflammation, with potential implications in a range of human chronic disorders including infectious diseases, cancer, and cardiovascular disease [4,6,7,8]. Recently, it was reported that the SNP rs2814778 on the Duffy gene may be associated with differences in the baseline levels of high-sensitivity C-reactive protein (hs-CRP), an important biomarker of chronic inflammation that predicts subsequent mortality and incident CVD among community-dwelling adults [9]. However, due to the historical Eurocentric bias of GWASs, there is limited information on the role of the Duffy-null allele in chronic disease outcomes. The purpose of this study was to investigate the association of Duffy receptor status and hs-CRP, and its effect on mortality and incident cardiovascular outcomes, in three contemporary, longitudinal cohort studies of self-identified Black adults recruited from the general population.

## 2. Methods

### 2.1. Study Design and Data Collection

**Figure 1:**
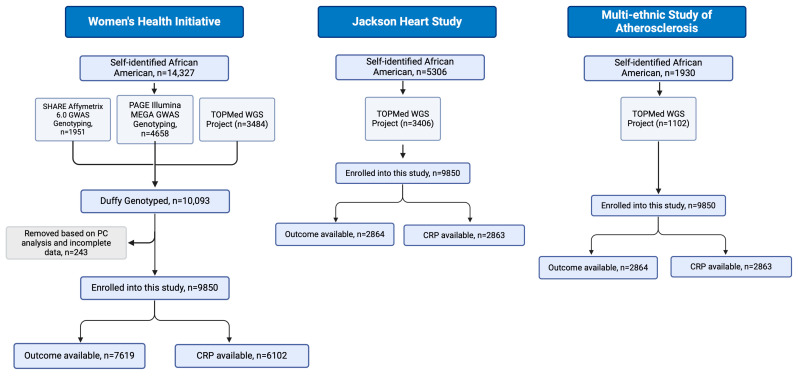
Flow diagram of enrolled participants.

This study was approved by the IRB at NewYork Presbyterian-Brooklyn Methodist Hospital. This study included a total of 14,358 self-identified Black or African American participants (9850 from Women’s Health Initiative (WHI), 1102 from Multi-Ethnic Study of Atherosclerosis (MESA), and 3406 from Jackson Heart Study (JHS)) whose Duffy-null genotype status (rs2814778) had been previously determined by either whole-genome sequencing or array-based genotyping and imputation (Figure 1).

The design and protocol of the prospective, multicenter Women’s Health Initiative longitudinal study has been previously described [10]. The WHI enrolled 161,808 postmenopausal women throughout the U.S. between 1993 and 1998 into an observational study or clinical trial(s) of hormone therapy, calcium/vitamin D supplementation, or dietary modification. Race/ethnicity was ascertained using self-report, categorized as (1) American Indian or Alaskan Native; (2) Asian or Pacific Islander; (3) Black or African American; (4) Hispanic/Latino; or (5) White (not of Hispanic origin). There were 14,327 self-reported Black or African American female participants, aged 50 to 79 at enrollment, from 40 U.S. clinical centers and enrolled between 1993 and 1998. All study sites obtained informed consent and institutional review board approval.

The design and protocol of the Jackson Heart Study (JHS) has been previously described [11,12]. Briefly, the JHS is an ongoing, prospective study in the United States that enrolled 5306 self-identified Black or African American participants (age range, 20–95 years), residing in the Jackson, Mississippi metropolitan area between September 2000 and March 2004. The JHS protocol and all data collection procedures were approved by the institutional review boards (IRBs) of the University of Mississippi Medical Center, Jackson State University, and Tougaloo College. All study participants provided written informed consent.

The design and protocol of the Multi-Ethnic Study of Atherosclerosis (MESA) (ClinicalTrials.gov: NCT00005487) has been previously described [13]. Briefly, we analyzed follow-up data over 15 years from the MESA: an ongoing multicenter, prospective population-based study in the United States that enrolled 6814 participants (age range, 45–84 years) between 2000 and 2002. In the MESA, approximately 38 percent of the recruited participants self-identified as White, 28 percent as Black, 22 percent as Hispanic, and 12 percent as Asian. The MESA was approved by the institutional review boards of each of the participating field sites in the United States (Wake Forest University, Winston-Salem, NC; Columbia University, New York City, NY; Johns Hopkins University, Baltimore, MD; University of Minnesota, Minneapolis, MD; Northwestern University, Evanston, IL; and University of California, Los Angeles, CA), and all participants provided written informed consent. All sites were compliant with the Health Insurance Portability and Accountability Act.

For all three cohorts, baseline sociodemographic and lifestyle characteristics and medical history were collected using standardized questionaries at the first visit. Blood pressure was measured by trained staff following a standardized protocol while participants were seated, on their right arm. Participants’ heights and weights were measured while they wore minimal clothing and no shoes, with values rounded to the nearest 0.1 cm and 0.5 kg, respectively. BMI was calculated as weight (kg) divided by height squared (m^2^). Smoking status was defined from a self-reported questionnaire administered at study entry. History of diabetes was ascertained by medical history according to 2003 American Diabetes Association Fasting Criteria in the MESA, 2010 American Diabetes Association Fasting Criteria in the JHS, and self-reported history in the WHI [14]. The income and education tiers were harmonized across the 3 cohorts as follows: Income was coded as 1—less than USD 5000 to 19,999; 2—USD 20,000 to 49,999; 3—USD 50,000 to 99,999; and 4—USD 100,000 or more. Education was coded as 1—no schooling to attainment up to grades 9–11; 2—completed high school or GED; and 3—some college but no degree to graduate or professional school.

### 2.2. Genotype Assessment of Duffy-Null Status, CRP Locus, and Principal Components of Genetic Ancestry

Rs2814778 SNP genotype CC (null mutation) was designated as Duffy-null, and rs2814778 genotype TT/CT was designated as Duffy-receptor-positive. Sequencing for the rs2814778 SNP genotype was performed by either whole-genome sequencing (WGS) (MESA, JHS, and a subset of 3484 WHI participants) or genome-wide genotyping with the Affymetrix SNP 6.0 or Illumina MEGA genotyping arrays and imputation from the NHLBI TOPMed reference panel for a subset of 6609 WHI participants.

Whole-genome sequencing with mean read coverage of 30X was performed by TOPMed sequencing centers in MESA, JHS, and WHI participants and is described in detail at https://www.nhlbiwgs.org/topmed-whole-genome-sequencing-methods-freeze-8 (accessed 1 August 2022). WGS data are available through restricted access via the NCBI database of Genotypes and Phenotypes (dbGaP). dbGaP accession numbers for the WHI (phs001237.v2.p1, phs000386.v8.p3, and phs000227.v5.p3), JHS (phs000964/phs002256.v1.p1), and MESA (phs001416.v1.p1) may require IRB and dbGaP Data Access Committee (DAC) approval. Imputation of rs2814778 genotype in the remaining WHI participants from the Affymetrix SNP 6.0 or Illumina MEGA genotyping arrays was performed using TOPMed reference panel. GWAS sequencing in the WHI SNP Health Association Resource (phs000386.v8.p3) was imputed using TOPMed reference panel; SNPs were filtered for minor allele frequency ≥ 1%, call rate ≥ 95%, and HWE *p*-value > 10^−6^, as well as exclusion of sites with invalid or mismatched alleles for the reference panel. Imputed values at the Duffy SNP (number of C alleles) were rounded to whole numbers as follows: 0 to 0.49 coded as 0, 0.5 to 1.49 coded as 1, and 1.5 to 2 coded as 2. Duffy-null status was coded as 1 if two C alleles at Duffy SNP were present and 0 if either one or no C alleles were present.

The Duffy-null genotype is highly correlated with African ancestry, and any results attributed to the null genotype may be due to other variants more common in those of African ancestry co-inherited with the null genotype. Thus, principal component analysis (PC 1 through 10) of sequencing data was utilized to control for genetic ancestry.

The 8 CRP SNP alleles (rs11265259, rs1800947, rs2211321, rs7551731, rs73024795, rs553202904, rs12734907, rs181704186) were genotyped in a similar manner to rs2814778 by either whole-genome sequencing (WGS) (MESA, JHS, and a subset of WHI participants) or genome-wide genotyping with the Affymetrix SNP 6.0 or Illumina MEGA genotyping arrays and imputation from the NHLBI TOPMed reference panel (for WHI).

### 2.3. Laboratory Measurements of hs-CRP

All serum samples were drawn at the baseline examination. In the JHS, fasting blood samples were drawn and processed using a standardized protocol and sent to Fairview University of Minnesota Medical Center (Minneapolis, MN, USA) for measurement of hs-CRP by the immunoturbidimetric CRP-Latex assay from (Kamiya Biomedical Company (Seattle, WA, USA) using a Hitachi 91l analyzer following the manufacturer’s protocol [15]. In the MESA, blood was drawn after a 12 h fast from participants, and aliquots (approximately 65 aliquots per participant) were prepared for central analysis and for storage at the University of Minnesota. CRP was measured using a BNII nephelometer (N high-sensitivity CRP; Dade Behring Inc., Deerfield, IL, USA) [16]. In the WHI, C-reactive protein was measured at baseline at the Advanced Research and Diagnostic Laboratory at the University of Minnesota Medical Center laboratory. Hs-CRP (mg/L) was measured in serum by immunoassay on a Roche Modular P Chemistry analyzer using a latex particle-enhanced immunoturbidimetric assay kit (Roche Diagnostics; Indianapolis, IN, USA) and read on the Roche Modular P Chemistry analyzer.

### 2.4. Adjudication of Clinical Outcomes

The primary endpoint was the incidence of all-cause mortality, which was verified in 12-month intervals by a telephone interview with participants, reviewing copies of death certificates and medical records for all hospitalizations, and annual National Death Index queries. Incident CVD events were examined as secondary endpoints. Incident coronary heart disease was defined as a composite of coronary heart disease mortality and non-fatal myocardial infarction in the JHS; coronary heart disease mortality and resuscitated cardiac arrest, definite or probable angina, and non-fatal myocardial infarction in the MESA; and acute MI requiring overnight hospitalization, silent MI determined by serial ECGs, or coronary heart disease death in the WHI.

Stroke (ischemic and hemorrhagic) was defined in each cohort using the following standardized criteria: In the JHS, stroke events were defined as a definite or probable hospitalized stroke from neuroimaging and autopsy based on the classification from the National Survey of Stroke [17]. The minimum criterion for probable or definite stroke was the rapid onset of neurological symptoms lasting >24 h or symptoms that led to death. The MESA used a standard adjudication protocol to classify events, as previously reported [18]. Stroke was defined as rapid onset of documented focal neurological deficits lasting 24 h or until death and, if <24 h, with imaging evidence (typically computed tomography or MRI) of a clinically relevant lesion. For the WHI, adjudicated stroke was classified as ischemic, hemorrhagic, or unknown stroke type. Extensive adjudication and subtyping procedures are described elsewhere [19].

Heart failure was defined in each cohort using the following standardized criteria: In the WHI, incident heart failure was defined as hospitalization for heart failure and adjudicated by trained physicians as definite or possible acute decompensated HF hospitalizations (regardless of ejection fraction) based on the algorithm used in the ARIC (Atherosclerosis Risk in Communities) study [20]. For the MESA, incident heart failure was defined as a composite of probable and definite HF. A positive event required symptoms of heart failure such as shortness of breath or oedema and a receipt for medical treatment for heart failure, all adjudicated by trained physicians. Definite HF also required one or more objective criteria, such as pulmonary oedema/congestion by chest X-ray; dilated ventricle or poor LV function by echocardiography or ventriculography; or evidence of LV diastolic dysfunction. In the JHS, heart failure events were defined as the occurrence of either inpatient or outpatient diagnoses of unspecified failure of the heart from the *International Classification of Diseases, Ninth Revision* (*ICD-9*) diagnosis code of 428.X in any position or 428 on a death certificate. The definition of heart failure also includes, but is not limited to, radiographic findings that were similar with congestive HF, increased venous pressure > 16 mm Hg, dilated ventricle/left ventricular function <40% from an ECG or multiple-gated acquisition, or autopsy finding of pulmonary edema.

In the JHS, the surveillance of mortality and incident coronary disease spanned from 2000 to 2018, whereas the surveillance of heart failure events spanned from 2005 to 2016. In the MESA, the surveillance period of all outcomes spanned from 2001 to 2018. In the WHI, followed-up occurred every 6 months, and annual in-clinic visits were required annually. At each 6-month follow-up, initial reports of outcome events were obtained using a self-administrated questionnaire. The surveillance period of all outcomes in the WHI spanned from 1993 to 2023.

### 2.5. Statistical Analysis

Continuous and categorical variables are presented as means with one standard deviation and were compared with a one-way *t*-test for means and a chi-square test for proportions. Duffy antigen status was found to be associated with diabetes on univariate analysis. Binary logistic regression was applied to explore the independent association of Duffy status and diabetes. Linear regression models were applied to determine the independent variables associated with serum hs-CRP levels in each cohort independently. hs-CRP values were log-transformed prior to modeling. The following demographic and clinical covariates were included in Model 1: age, sex, diabetes, systolic blood pressure, active smoking, income, education, BMI, PC1–10, sequencing study variable (imputed vs. sequenced in WHI only), and Duffy status. These demographic and clinical covariables were selected as these factors are known to be correlated with the levels of hs-CRP. The Duffy gene and hs-CRP gene are in relative proximity on chromosome 1. To account for the possibility of long-range linkage disequilibrium, sensitivity analysis was carried out in Model 2 which included all covariates from Model 1 with the addition of the genotypes for eight *CRP* locus SNPs that are known to influence the circulating levels of CRP: rs11265259, rs1800947, rs2211321, rs7551731, rs73024795, rs553202904, rs12734907, and rs181704186 [21]. The resultant estimated regression coefficient (B) and corresponding standard error for the two successive models from each cohort were then meta-analyzed utilizing a random-effects continuous outcome model.

Next, binary logistic regression modeling was applied to explore the association between Duffy-null status and history of diabetes in each cohort independently. Model 1 was a univariate analysis of Duffy null (coded recessively) only, and Model 2 was adjusted for age, sex, and PC1–10, and Duffy-null status.

Multivariable Cox proportional hazard regression was used to determine the independent predictors of the primary (mortality) and secondary (incident CVD event) outcomes. The following demographic and clinical covariates were included in three successive models for the primary and secondary outcomes: Model 1—age, sex, Duffy status (coded recessively), principal component 1–10, and sequencing study variable (imputed vs. sequenced in WHI only); Model 2—covariables of Model 1 plus systolic blood pressure, history of diabetes, active smoking status, body mass index, income tier, and education tier; and Model 3—Model 2 plus hs-CRP. The resultant log(hazard ratio), or estimated regression coefficient (B), and corresponding standard error for the three successive models from each cohort were then meta-analyzed utilizing a generic inverse variance-weighted average random-effects model.

Exploratory analysis of cause-specific mortality was performed in the WHI and MESA cohorts (cause-specific events are not available in the JHS) utilizing similar multivariable Cox proportional hazard regression models. The following cause-specific mortality classifications were considered: death from coronary heart disease, stroke, infectious disease including COVID-19, cancer, and Alzheimer/dementia.

The number of included participants for each of the analyses differed from one another because of missing datapoints.

The significance level for all analyses was set at *p* < 0.05 (two-sided). All analyses and figures were performed and generated with IBM SPSS Statistics for Mac, version 29 (IBM Corp., Armonk, NY, USA), BioRender software (https://biorender.com/ (accessed on 26 September 2024)), and Review Manager (RevMan) (2014) Version 5.3., Copenhagen, Denmark: The Nordic Cochrane Centre, The Cochrane Collaboration. The linkage disequilibrium heatmap plot was generated using the SRPLOT online tool (http://www.bioinformatics.com.cn/srplot (accessed on 15 June 2024).

### 2.6. Replication Analysis in United Kingdom Biobank Repository

Replication analysis for long-term outcomes was performed in a cohort of self-reported Black participants of the United Kingdom Biobank Repository for whom the genotype at rs2184778 was known through UK10k/HRC imputed data, as previously described. Multivariable Cox proportional hazard regression was used to determine the independent predictors of all-cause mortality. The following demographic and clinical covariates were included in three successive models for the primary and secondary outcomes: Model 1—age, male sex, Duffy status (coded recessively), and principal components 1–10; Model 2—variables of Model 1 + systolic blood pressure, history of diabetes, smoking status, body mass index, and education tier; and Model 3—Model 2 + hs-CRP.

## 3. Results

From the WHI, JHS, and MESA cohorts, 14,358 self-identified Black or African American participants (9850 women from WHI, 3406 from JHS, and 1102 from MESA) for whom genotype data at the Duffy SNP were available were included in these analyses. Baseline characteristics, by Duffy receptor status and cohort, are displayed in Table 1. In univariate analyses, participants in the Duffy-null group were younger and had lower income and education, higher hs-CRP, and a higher prevalence of diabetes. Additional adjustment for age, sex, and PCs showed that the association between Duffy-null status and history of diabetes was largely due to confounding by genetic ancestry (Table 2b).

### 3.1. Association of Duffy Null with hs-CRP Levels

**Figure 2:**
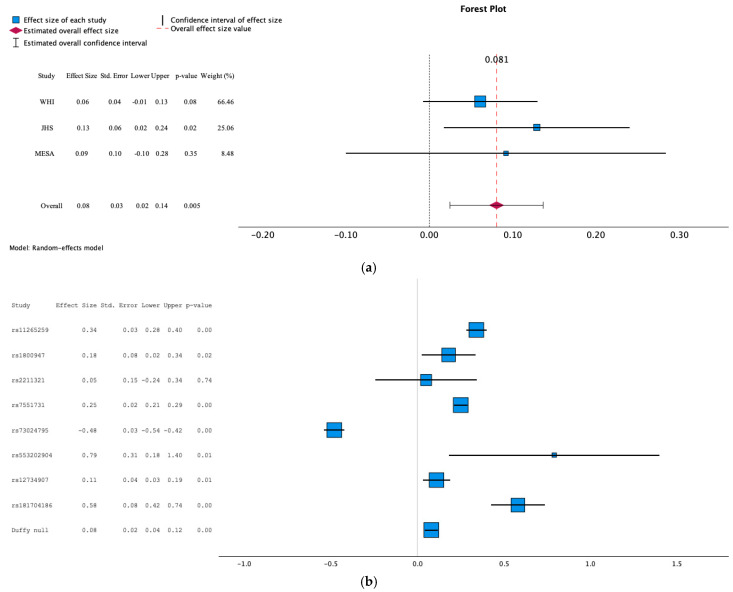
(**a**) Meta-analysis of effect of Duffy null on hs-CRP. Fully adjusted for age, sex, PC1–10, study type (sequencing vs. imputed), systolic blood pressure, diabetes, smoking, body mass index, income and education tier, and 8 CRP locus alleles. (**b**) Effect sizes of 8 CRP locus alleles and Duffy-null status from Model 2 on hs-CRP levels. Fully adjusted for age, sex, PC1–10, study type (sequencing vs. imputed), systolic blood pressure, diabetes, smoking, body mass index, and income and education tier.

In all three cohorts, the Duffy-null status was strongly associated with higher hs-CRP levels (meta-analysis *p* = 2.62 × 10^−9^) (Table 2a). Using genotype data from the largest WHI cohort, the Duffy-null variant rs2814478 showed varying degrees of linkage disequilibrium with eight *CRP* locus alleles previously reported to be independently associated with hs-CRP. These eight *CRP* locus alleles were identified in a partially overlapping sample to those utilized here [21]. The pairwise r2 with rs2814778 ranged from 0.002 for rs553202904 to 0.24 for rs12734907 (Supplemental Figure S1). Upon additionally adjusting for these eight *CRP* locus alleles, the Duffy null–hs-CRP association was greatly attenuated with effect sizes reduced by ~60% (Table 2a). Meta-analysis of the *CRP* locus-adjusted results showed a marginally significant association (*p* = 0.005) of the Duffy-null genotype with higher hs-CRP levels (Figure 2a) [21]. The magnitude and direction of the effect size of Duffy null and the eight *CRP* locus alleles meta-analyzed across the three cohorts are displayed in Figure 2b.

### 3.2. Association of Duffy Null with Risk of Mortality

**Figure 3:**
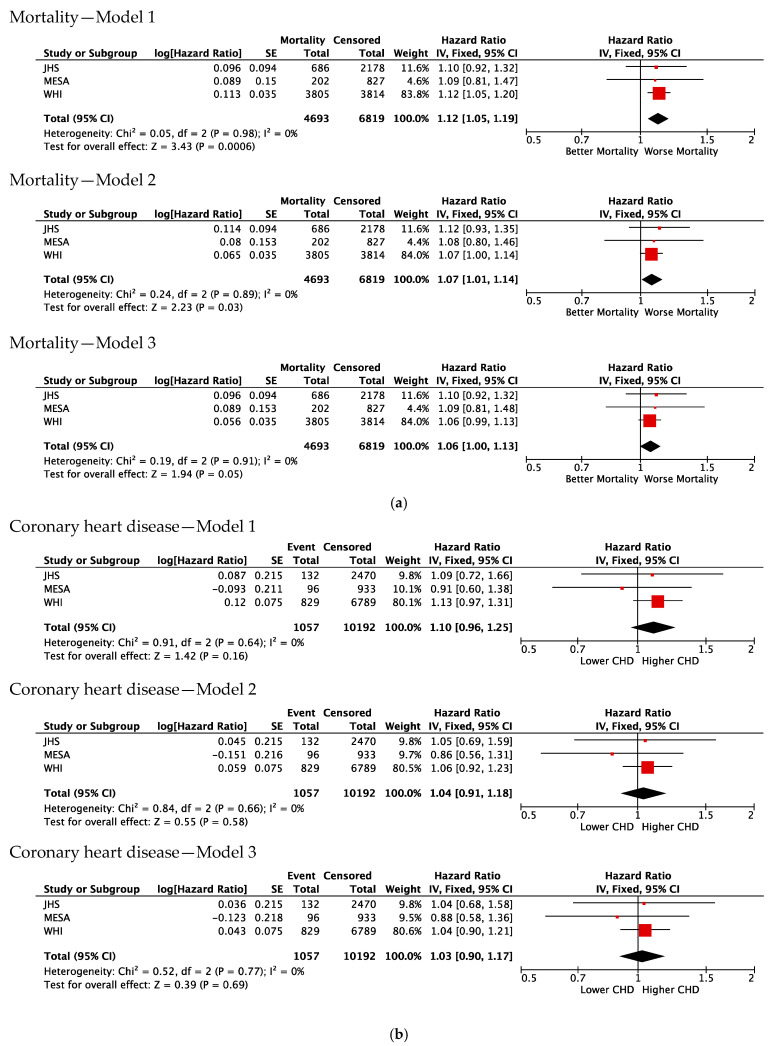

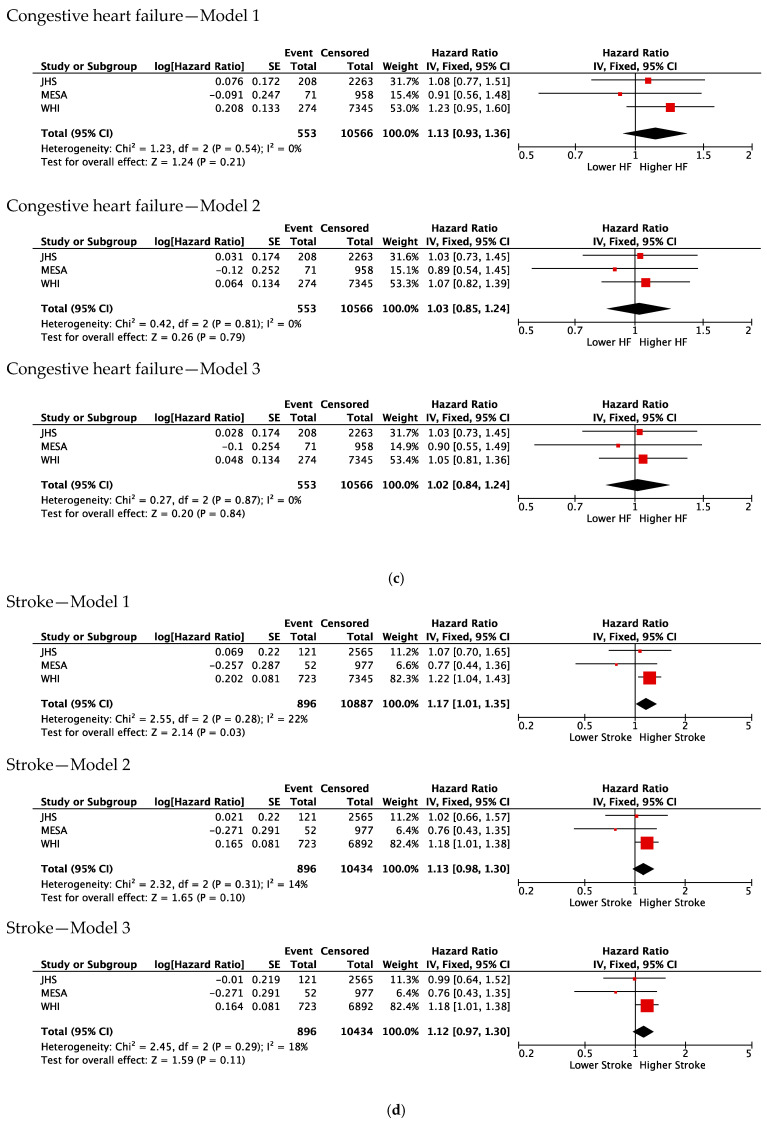
Model 1 = age, sex, and PC1–10; Model 2 = Model 1 + systolic blood pressure, diabetes, smoking, body mass index, and income and education tier; Model 3 = Model 2 + hs-CRP.

The clinical outcome analysis in the WHI, JHS, and MESA included 11,512 Black participants. The primary endpoint of all-cause mortality occurred in 4693 or 40.7% of participants, with a median follow-up of 18.3 years. The meta-analysis of Cox regression modeling from the three cohorts for all-cause mortality was significant in Model 1 (*p* = 0.0006), attenuated by traditional CV risk factors in Model 2 (*p* = 0.03), and was further attenuated by additional adjustment for hs-CRP in Model 3 (*p* = 0.06) (Figure 3a). However, replication analysis of the Duffy-null status and all-cause mortality performed in the UKBB registry did not demonstrate a concordant signal with that seen in the TOPMed cohorts (i.e., HR < 1, Table 3).

We performed additional exploratory analysis of cause-specific mortality in the WHI and MESA cohorts (cause-specific events are not available in JHS). Cox regression modeling from the WHI and MESA for all-cause specific mortality is shown in Table 4a,b, respectively. The association between the Duffy-null status and cause-specific mortality was strongest for coronary heart disease in both cohorts. The effect size was attenuated once adjusted for baseline risk factors and hs-CRP in the WHI, whereas the association in the MESA persisted (*p* = 0.17, and 0.02, respectively). There was no evidence of an association between the Duffy-null status and cause-specific mortality from stroke, infectious disease, cancer, or Alzheimer/dementia in either minimally or fully adjusted regression models.

### 3.3. Association of Duffy Null with Incident CVD

The secondary endpoint of incident coronary heart disease occurred in 1057 participants, or 9.4% of the TOPMed cohort. The meta-analysis of Cox regression modeling from the three cohorts for incident coronary heart disease was not significant in Model 1 (*p* = 0.16) and attenuated further in Model 2 and 3 (*p* = 0.58 and *p* = 0.69) (Figure 3b). The secondary endpoint of incident heart failure occurred in 553 participants, or 4.9% of the cohort. The meta-analysis of Cox regression modeling from the three cohorts for incident heart failure was not significant in Model 1 (*p* = 0.21) and further attenuated in Models 2 and 3 (*p* = 0.58 and *p* = 0.69) (Figure 3c). The secondary endpoint of incident stroke occurred in 896 participants, or 7.9% of the cohort. The meta-analysis of Cox regression modeling from the three cohorts for incident stroke was significant in Model 1 (*p* = 0.03) but became attenuated in Models 2 and 3 (*p* = 0.10 and *p* = 0.11) (Figure 3d).

## 4. Discussion

The major findings from this analysis of Black participants of three longitudinal U.S.-based cohorts are as follows: (1) The Duffy-null receptor status was strongly associated with higher hs-CRP, but the association was partially attenuated after conditioning on *CRP* locus alleles known to be associated with changes in the expression level of CRP. (2) There was no robust evidence of an association between the Duffy-null status and incident mortality or CVD events during follow-up after adjusting for measured CRP levels and other known risk factors.

CRP is a complex, multifactorial phenotype influenced by both non-genetic/environmental factors such as age, sex, diabetes, systolic blood pressure, smoking, socioeconomic status, and BMI and genetic factors such as *CRP* locus alleles [22,23,24,25,26]. Prior genetic studies have implicated Duffy-null status as a possible genetic contributor to hs-CRP [9,27]. Given the proximity of the Duffy gene and CRP locus on chromosome 1, along with the extensive long-range admixture linkage disequilibrium surrounding rs2814778, it is important to assess the relationship of Duffy receptor status to hs-CRP and other immune-related biomarkers and subsequent long-term cardiovascular outcomes in the context of other genetic variants at the *CRP* locus that are known to strongly influence hs-CRP levels. After conditioning on eight *CRP* locus alleles, the association of Duffy-null status with higher hs-CRP levels was considerably attenuated but remained statistically significant (*p* = 0.005). These eight *CRP* variants were identified in a whole-genome-sequencing-based pooled ancestry GWAS of CRP that included the TOPMed cohorts, including the JHS, WHI, and MESA [21]. The prior TOPMed pooled ancestry CRP GWAS, which coded the Duffy-null rs2814778 allele additively, similarly did not identify the Duffy allele as a genome-wide significant signal while conditioning on all eight CRP variants (*p* = 0.0002) [21].

Taken together, these results suggest that the apparent association between the Duffy-null status and higher CRP level is largely due to partial linkage disequilibrium with other CRP-associated variants spanning the CRP locus on chromosome 1q23. The population genetic complexity of this region, including the strong admixture LD, makes it difficult to disentangle the contribution of the two loci from a purely statistical standpoint [28]. Functionally, the loss of expression of the Duffy receptor protein on erythroid cells may modulate circulating levels of various cytokines/chemokines leading to increased chronic inflammation and CRP [29]. Alternatively, rs2814778 might tag other undiscovered variants more common in African reference populations that directly regulate CRP expression [21]. Our analysis does not the exclude the possibility that additional CRP locus alleles may be contributing to the observed associations of Duffy-null status with CRP; other GWASs have identified an even larger number of distinct variants associated with CRP at the *CRP* locus [30].

The relationship between the Duffy-null variant and CRP is further complicated by the association of Duffy null with lower circulating levels of neutrophils. Higher neutrophil counts are often a marker of inflammation and are generally associated with worse clinical outcomes [4,31]. Paradoxically, the correlation of Duffy null and higher hs-CRP suggests that the utilization of white blood cell counts as a marker of inflammation may require the consideration of Duffy status since the effect modification of Duffy status on neutrophil-to-lymphocyte levels on CVD outcomes has been demonstrated in the JHS [5].

Regardless of the mechanism or causal genetic variant(s) underlying the association of Duffy-null status with higher hs-CRP, given (a) the high prevalence of Duffy null among Black individuals and (b) the higher incidence of mortality and CVD among Black adults, from a public health perspective, it is important to understand whether Duffy null is a risk factor for mortality or CVD outcomes among Black adults [1]. In minimally adjusted analyses, Duffy-null status was associated with a higher risk of all-cause mortality, CHD-specific mortality, and incident stroke. However, Duffy receptor status was no longer a statistically significant predictor of these outcomes following the adjustment of traditional cardiovascular risk factors and hs-CRP. This suggests the possibility that the lack of the erythroid Duffy receptor in Black individuals may have some influence on mortality or CVD outcomes that is at least partially mediated through chronic inflammation [32,33]. Other changes in inflammatory proteins and cell types have also been reported in Duffy-null carriers, which could contribute to a proinflammatory state [33]. On the other hand, replication analysis carried out in the UKBB cohort did not demonstrate a concordant signal with that seen in the TOPMed cohorts, which may be due to differences related to a variety of factors including the sociodemographic, geographic, or environmental background of the study participants, study design and recruitment, or statistical power of the UKBB replication sample. Factors unrelated to inflammation, including healthcare disparities, as previously reported for Duffy-null carriers, could also influence any Duffy-null-associated risk of mortality [34]. Therefore, additional research addressing the role of the erythroid ACKR1 receptor in inflammation and atherosclerosis as well as larger observational studies conducted in populations with a high prevalence of the Duffy-null allele will be required to define the relevance of this common polymorphism with respect to chronic disease and mortality.

### Limitations

We recognize important limitations to our study design, observations, and conclusions. The first is the presence of recruitment/selection bias that may limit generalizability to the U.S. population (e.g., MESA inclusion criteria required asymptomatic individuals free of CVD) [35]. Second, only baseline demographic and risk factors were considered in our analysis; thus, it is unclear whether certain risk factors progressed or regressed over time in the study participants. Third, the absolute mortality difference between Duffy groups is small (0.5 to 1 percent), suggesting our analysis may be inadequately powered in its current design.

## 5. Conclusions

Hs-CRP was significantly higher in the Duffy-null group, but this association was largely attenuated by CRP SNP alleles in linkage disequilibrium to the Duffy gene. Duffy-null status was associated with a slightly higher incidence of all-cause mortality and stroke in Black adults, but these associations were not significant after adjusting for traditional CVD risk factors including hs-CRP. Taken together, these results suggest a possible immunomodulatory role of the Duffy-null phenotype, but additional research is required. Given the ongoing health inequities in cardiovascular outcomes in the Black population, more needs to be done to understand both the determinants driving such outcomes so that personalized therapies and risk modification schema may be developed and implemented to addresses these disparities.

## Figures and Tables

**Table 1:** Baseline characteristics by Duffy antigen receptor status.

WHI			
	Duffy-antigen-receptor-negative (n = 5959)	Duffy-antigen-receptor-positive (n = 3891)	*p*-value
**Demographics**			
Age (years)	61.2 ± 7.0	62.0 ± 7.2	<0.001
Males (%)	-	-	-
Income tier	2.0 ± 0.8	2.1 ± 0.8	<0.001
Education tier	1.6 ± 0.7	1.7 ± 0.6	<0.001
**Clinical variables**			
Systolic blood pressure	132.3 ± 17.8	131.7 ± 17.8	0.31
Diabetes	917 (15)	531 (14)	0.02
Current smoker	661 (11)	429 (11)	0.97
BMI (kg/m^2^)	31.2 ± 6.7	31.1 ± 6.7	0.54
hs-CRP (mg/L)	6.6 ± 7.9	5.6 ± 8.3	<0.001
**JHS**			
	Duffy-antigen-receptor-negative (n = 2389)	Duffy-antigen-receptor-positive (n = 1017)	*p*-value
**Demographics**			
Age (years)	54.7 ± 12.8	56.0 ± 12.8	0.04
Males (%)	890 (37)	374 (37)	0.79
Income tier	2.7 ± 1.0	2.8 ± 1.0	0.10
Education tier	1.4 ± 0.8	1.5 ± 0.7	0.008
**Clinical variables**			
Systolic blood pressure	127.4 ± 17.8	126.5 ± 16.5	0.18
Diabetes	596 (25)	235 (23)	0.25
Current smoker	318 (13)	127 (12)	0.51
BMI (kg/m^2^)	31.9 ± 7.2	31.7 ± 7.5	0.72
hs-CRP (mg/L)	5.6 ± 10.8	4.4 ± 6.9	<0.001
**MESA**			
	Duffy-antigen-receptor-negative (n = 675)	Duffy-antigen-receptor-positive (n = 427)	*p*-value
**Demographics**			
Age (years)	60.6 ± 9.5	61.4 ± 9.7	0.49
Males (%)	317 (47)	201 (47)	0.97
Income tier	2.3 ± 0.9	2.4 ± 0.9	0.07
Education tier	2.6 ± 0.7	2.6 ± 0.6	0.10
**Clinical variables**			
Systolic blood pressure	130.4 ± 21.1	128.9 ± 19.5	0.74
Diabetes	101 (15)	75 (18)	0.24
Current smoker	124 (18)	74 (17)	0.66
BMI (kg/m^2^)	29.9 ± 5.4	30.0 ± 5.4	0.24
hs-CRP (mg/L)	4.0 ± 5.4	3.6 ± 6.2	0.02

BMI—body mass index; hs-CRP—high-sensitivity c-reactive protein.

**Table 2:** (**a**) Association of Duffy null with hs-CRP with and without conditioning for 8 CRP alleles. (**b**) The association of Duffy null and diabetes.

(**a**)					
		**Estimate (B)**	**Std. Error**		***p*-Value**
WHI (n = 6102)	Model 1 *	0.189	0.031		1.09 × 10^−9^
	Model 2 **	0.061	0.035		0.08
JHS (n = 2863)	Model 1	0.293	0.049		3.38 × 10^−9^
	Model 2	0.129	0.057		0.02
MESA (n = 830)	Model 1	0.201	0.085		0.02
	Model 2	0.092	0.098		0.35
Meta-analysis	Model 1	0.18	0.069		2.62 × 10^−9^
	Model 2	0.08	0.03		0.005
(**b**)					
		**Estimate (B)**	**Standard Error**	**Hazard Ratio**	***p*-Value**
WHI (n = 10,083)	Model 1 *	0.140	0.059	1.50 [1.03, 1.29]	0.02
	Model 2 **	0.085	0.063	1.09 [0.96, 1.23]	0.18
JHS (n = 3406)	Model 1	0.101	0.088	1.11 [0.93, 1.31]	0.25
	Model 2	0.035	0.099	1.04 [0.85, 1.25]	0.72
MESA (n = 1102)	Model 1	−0.195	0.167	0.82 [0.59, 1.14]	0.24
	Model 2	−0.246	0.175	0.78 [0.55, 1.10]	0.16

(a) Dependent variable: Natural log(hsCRP). * Model 1 adjusted for age, sex, diabetes, systolic blood pressure, current smoking, income, education, BMI, and PC1–10. ** Model 2 adjusted for Model 1 independent variables + rs11265259, rs1800947, rs2211321, rs7551731, rs73024795, rs553202904, rs12734907, and rs181704186. (b) * Model 1—unadjusted. ** Model 2—adjusted for age, sex, and PC1–10.

**Table 3:** Multivariate regression modeling of Duffy null for all-cause mortality in UKBB.

	Hazard Ratio [95% CI]	*p*-Value
Model 1 (n = 8821, n = 529 events)	0.81 [0.61, 1.06]	0.12
Model 2 (n = 6973, 358 events)	0.75 [0.54, 1.05]	0.09
Model 3 (n = 6527, 340 events)	0.73 [0.53, 1.01]	0.06

Covariates for Model 1 = age, sex, and PC1–10; Model 2 = Model 1 + systolic blood pressure, diabetes, smoking, body mass index, and education tier; Model 3 = Model 2 + hs-CRP.

**Table 4:** (**a**) Multivariate regression modeling of Duffy null for cause-specific mortality in WHI. (**b**) Multivariate regression modeling of cause-specific mortality in MESA.

(**a**)										
	**Coronary Heart Disease** **(1025 Events, 6592 Censored)**		**Stroke** **(331 Events, 7282 Censored)**		**Infectious Disease** **(221 Events, 7359 Censored)**		**Cancer** **(965 Events, 6646 Censored)**		**Alzheimer/Dementia** **(349 Events, 6812 Censored)**	
	Hazard Ratio [95% CI]	*p*-value	Hazard Ratio [95% CI]	*p*-value	Hazard Ratio [95% CI]	*p*-value	Hazard Ratio [95% CI]	*p*-value	Hazard Ratio [95% CI]	*p*-value
Model 1	1.20 [1.05, 1.38]	0.007	1.11 [0.88, 1.40]	0.38	1.15 [0.87, 1.53]	0.33	1.01 [0.88, 1.15]	0.89	0.97 [0.78, 1.21]	0.81
Model 2	1.11 [0.98, 1.28]	0.11	1.05 [0.84, 1.33]	0.66	1.06 [0.80, 1.42]	0.66	0.99 [0.86, 1.14]	0.94	0.94 [0.76, 1.18]	0.64
Model 3	1.09 [0.96, 1.25]	0.17	1.05 [0.83, 1.33]	0.68	1.04 [0.78, 1.39]	0.76	0.99 [0.86, 1.13]	0.89	0.96 [0.76, 1.20]	0.72
(**b**)										
	**Coronary Heart Disease** **(54 Events, 975 Censored)**									
	Hazard Ratio [95% CI]					*p*-value				
Model 1	2.11 [1.14, 3.92]					0.02				
Model 2	2.04 [1.08, 3.80]					0.03				
Model 3	2.14 [1.13, 4.04]					0.02				

Model 1 = age, sex, and PC1–10; Model 2 = covariates of Model 1 + systolic blood pressure, diabetes, smoking, body mass index, and income and education tier; covariates of Model 3 = Model 2 + hs-CRP.

## Data Availability

Data may be obtained from a third party and are not publicly available. The datasets supporting the conclusions of this article originate from three separate third-party sources. Restrictions apply to the availability of the data, which were used under fully executed Data Materials Distribution Agreement and Data Use Agreements. Data from the Jackson Heart Study are available with a request to access at https://www.jacksonheartstudy.org/Research/Study-Data/Data-Access, accessed on 9 October 2024. Data from the Multi-Ethnic Study of Atherosclerosis are available with a request to access at https://www.mesa-nhlbi.org/Publications.aspx, accessed on 9 October 2024. Data from the Women’s Health Initiative are available with permission at https://www.whi.org/md/working-with-whi-data, accessed on 9 October 2024.

## Supplementary Materials

The following supporting information can be downloaded at https://www.mdpi.com/article/10.3390/genes15111382/s1, Figure S1: Linkage Disequilibrium Heatmap.
