# Supplementary figures and images for: The Relationship of Duffy Gene Polymorphism with High-Sensitivity C-Reactive Protein, Mortality, and Cardiovascular Outcomes in Black Individuals
Source: Genes (Basel). 2024 Oct 27;15(11):1382. doi: 10.3390/genes15111382 (PMC11594091; doi:10.3390/genes15111382)

# Linkage Disequilibrium Heatmap

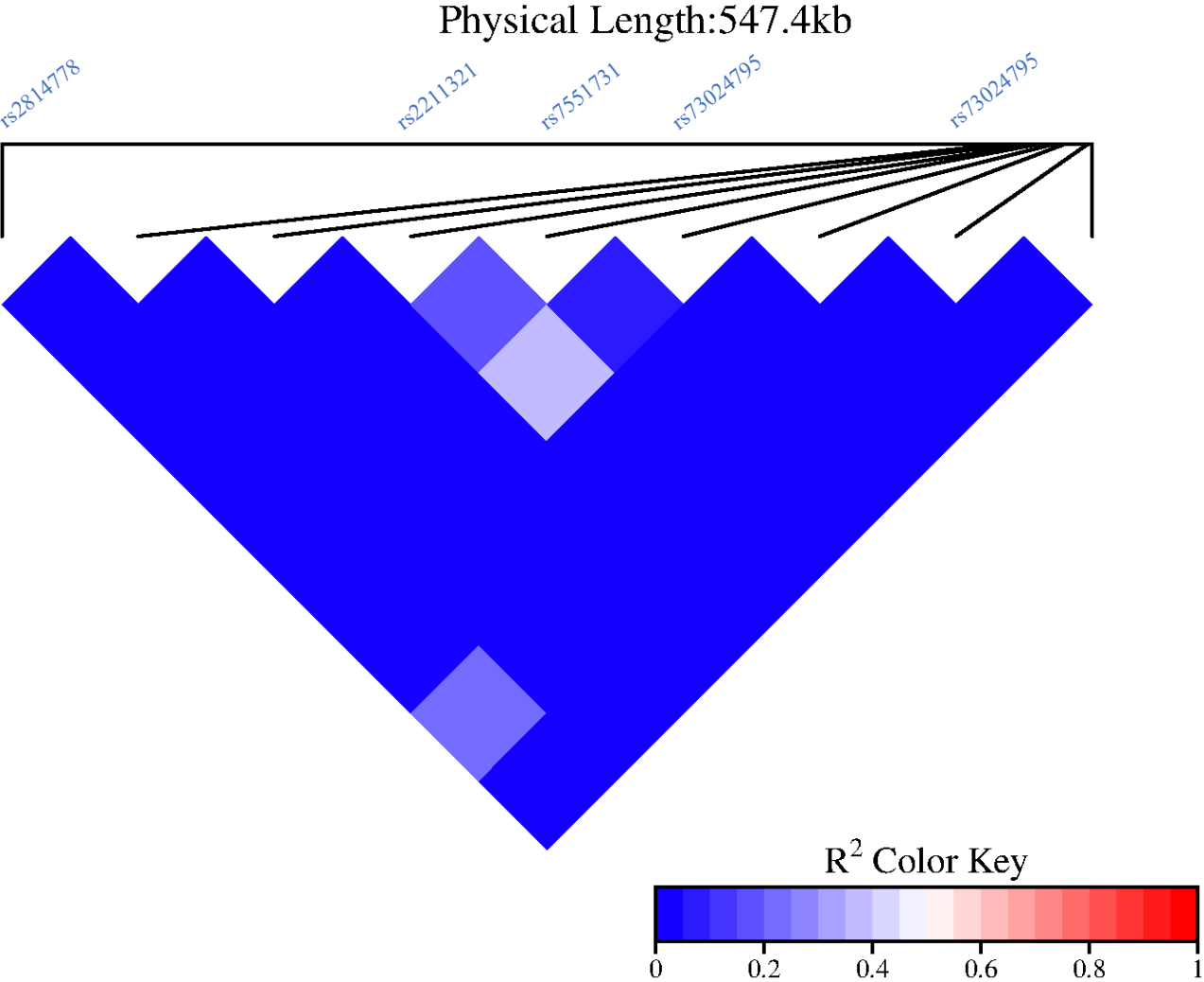

Supplement: Supplementary file 1 [file genes-15-01382-s001.zip › genes-3277487-supplementary.pdf]
